# Kynurenine pathway metabolites and therapeutic response to olanzapine in female patients with schizophrenia: A longitudinal study

**DOI:** 10.1111/cns.13895

**Published:** 2022-06-29

**Authors:** Xiaoni Guan, Jing Xu, Meihong Xiu, Xirong Li, Haixia Liu, Fengchun Wu

**Affiliations:** ^1^ Peking University HuiLongGuan Clinical Medical School Beijing HuiLongGuan Hospital Beijing China; ^2^ Qingdao Mental Health Center Qingdao Medical University Qingdao China; ^3^ Department of Psychiatry, Shandong Mental Health Center Shandong University Jinan China; ^4^ Department of Psychiatry The Affiliated Brain Hospital of Guangzhou Medical University Guangzhou China; ^5^ Guangdong Engineering Technology Research Center for Translational Medicine of Mental Disorders Guangzhou China

**Keywords:** kynurenine, metabolite, olanzapine, schizophrenia, therapeutic response

## Abstract

**Aim:**

A metabolomics approach has recently been used to identify metabolites associated with response to antipsychotic treatment. This study was designed to identify the predictive biomarkers of response to olanzapine monotherapy using a metabolomics‐based strategy.

**Methods:**

Twenty‐five first‐episode and drug‐naïve female patients with schizophrenia were recruited and treated with olanzapine for 4 weeks. Psychiatric symptoms were assessed using the Positive and Negative Syndrome Scale (PANSS) at baseline and 4‐week follow‐up.

**Results:**

Positive subscore, general psychopathology subscore, and PANSS total score were significantly decreased after treatment. An ultra‐performance liquid chromatography‐mass spectrometry (UPLC‐MS)‐based metabolomics approach identified 72 differential metabolites after treatment. In addition, the baseline levels of methyl n‐formylanthranilate (MNFT) were correlated with the rate of reduction in the positive subscore or PANSS total score. However, increase in MNFT after treatment was not associated with the rate of reduction in the PANSS total score or its subscores. Subsequent regression analysis revealed that the baseline MNFT levels predicted the treatment outcomes after olanzapine monotherapy for 4 weeks in patients with schizophrenia.

**Conclusions:**

Our study results suggest that the baseline MNFT levels in the kynurenine pathway of tryptophan metabolism may be predictive of the treatment response to olanzapine in schizophrenia.

## Graphical abstract

Methyl n‐formylanthranilate predict the therapeutic response to olanzapine in schizophrenia.

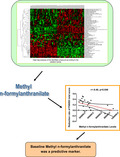

## 1 INTRODUCTION

Schizophrenia (SZ) is one of the most common types of mental illness, which exhibits heterogeneity in psychotic symptoms, clinical response to antipsychotic medication, and side effects.^1^, ^2^ Atypical antipsychotics are now considered to be the first‐line treatment of SZ.^3^ However, there are still some patients with SZ who do not respond adequately to antipsychotic medication because the precise mechanism is not fully understood. There is a need to identify and validate biomarkers for monitoring the therapeutic response in SZ patients.

Studies have shown that the clinical response to antipsychotic medication in patients with SZ varies substantially among individuals and with the type of antipsychotic as well as between the sexes. There is strong evidence that women with SZ have significantly higher response rates to antipsychotics than men with SZ.^4^, ^5^ Studies have also shown that olanzapine appears to be slightly more effective than other antipsychotics in the treatment of SZ.^6^ Moreover, olanzapine was reported to have the lowest risk of all‐cause discontinuation in the European First Episode Schizophrenia Trial (EUFEST) study and the longest withdrawal time in the Clinical Antipsychotic Trials of Intervention Effectiveness (CATIE) schizophrenia trial.^7^, ^8^ However, recent epidemiological data have shown that approximately 30% of patients with SZ do not respond to olanzapine and develop treatment‐resistant SZ over the course of the illness.^9^ In particular, it should be noted that approximately 50% of SZ patients treated with olanzapine gain a clinically significant amount of body weight. Predictive biomarkers can be used to improve the clinical management of SZ patients treated with olanzapine. Thus, there is an urgent need to identify novel biomarkers to monitor the efficacy of antipsychotics in patients with first‐episode SZ.

Several of the previous biomarkers for predicting the individual treatment response to antipsychotics were based on the molecular pathological mechanism of SZ and the biochemical pathway,^10^, ^11^, ^12^ such as brain‐derived neurotrophic factor (BDNF), 5‐hydroxytryptamine receptor 2C (HTR_2_C), serotonin (5‐HT2A (5‐HT_2A_), leptin, and fat mass and obesity‐associated gene (FTO).^13^, ^14^, ^15^, ^16^ However, SZ is a complex disorder with dysregulation of multiple biomarker pathways.^17^ Furthermore, SZ is highly heterogeneous and its pathogenesis is related to several risk factors, including genetic and environmental factors. Notably, multiple molecules in the tryptophan metabolic pathway have also been found to be associated with the therapeutic response and may be potential targets for drug discovery.^18^, ^19^ For example, a recent meta‐analysis by Cao et al. demonstrated that the levels of kynurenine (KYN) in the cerebrospinal fluid and plasma were lower in patients with SZ. In addition, KYN levels were elevated after treatment with antipsychotics relative to the baseline levels.^20^

In the previous decade, a metabolomics approach has provided comprehensive insights into the pathophysiological status of a disease at a certain time.^21^ Metabolomics measures the end products of the complex interrelationships between multiple proteins, signaling cascades, and cellular environments in biological samples (e.g., tissues, blood, urine, cerebrospinal fluid),^22^ and is divided into two distinct approaches: untargeted metabolomics and targeted metabolomics. The untargeted metabolomics approach is a comprehensive analysis of all measurable analytes in a sample and must be combined with advanced chemometric techniques to reduce the large data set to smaller manageable signal sets.^23^ Specifically, a high‐throughput metabolomics approach is a better systemic tool for identifying potential biomarkers and has been used to investigate the alterations in metabolites after treatment with antipsychotics.^24^

Various metabolites have been identified that can be linked to predict response to antipsychotic medication in patients with SZ.^21^, ^25^, ^26^, ^27^ A recent study found that specific lipid profiles could be used to predict response to different antipsychotic drugs using lipid metabolomics.^28^ Our previous study, through an untargeted metabolomics approach, identified 72 differential metabolites, which were found to be associated with weight gain after 1 month of treatment with olanzapine in patients with SZ.^29^ Another study by Ying et al. identified 13 metabolites using metabolomics techniques and reported that after treatment with olanzapine for 4 weeks in women with SZ, eight of these metabolites showed a significant change.^30^ However, until now, no study has investigated whether the baseline levels of KYN pathway metabolites can predict the response to olanzapine monotherapy in first‐episode drug‐naïve (FEDN) patients with SZ using an untargeted metabolomics approach based on ultra‐performance liquid chromatography‐mass spectrometry (UPLC‐MS).

Olanzapine is a second‐generation atypical antipsychotic with proven efficacy in the treatment of schizophrenia.^31^ Currently, it is one of the most popular antipsychotics and is widely used in both first‐episode and chronic cases.^32^, ^33^, ^34^ Therefore, in the present study, olanzapine was used to treat patients with FEDN SZ. Previous studies have demonstrated sex differences in the illness onset and degree of treatment response in first‐episode patients with SZ.^35^, ^36^, ^37^ To minimize the impact of confounding factors, in this study, we recruited only female FEDN patients who did not smoke and were not alcohol dependent. We hypothesized that after 4‐week olanzapine monotherapy, the levels of the baseline KYN metabolites in SZ may be different between those who respond poorly and those who respond well. To test this hypothesis, we used the UPLC‐MS‐based untargeted metabolomics platform to identify the differential compounds associated with the KYN metabolic pathway after treatment with olanzapine for 4 weeks.

## 2 METHODS

### 2.1 Participants

This open‐label study was carried out at Beijing Huilongguan Hospital. Twenty‐five female FEDN patients with SZ were diagnosed according to the Diagnostic and Statistical Manual of mental disorders‐IV (DSM‐IV) criteria and confirmed by the Structured Clinical Interview of DSM‐IV Axis I disorder (SCID). Inclusion criteria were as follows: (1) Han Chinese women; (2) 18–45 years old; and (3) drug‐naïve or previous antipsychotic treatment <2 weeks. Exclusion criteria were as follows: (1) any other major Axis I disorder; (2) organic brain disease, traumatic brain injury, or other systemic disease; (3) electroconvulsive therapy in the previous 6 months; (4) pregnant or lactating women; and (5) smoking, alcohol, or substance abuse. A complete set of physical examination, medical history, and laboratory tests was performed in all patients to exclude serious physical conditions.

The current study was conducted in accordance with the Declaration of Helsinki. This study was approved by the Institutional Review Board of Beijing Huilongguan Hospital, and each patient provided written informed consent.

### 2.2 Study procedures

This was a longitudinal, observational study in which FEDN patients were treated with flexible‐dose olanzapine monotherapy for 1 month. The dose of olanzapine ranged from 10 to 30 mg/day according to an experienced psychiatrist's judgment. All patients were hospitalized throughout the clinical study. Nurses monitored adherence to olanzapine treatment.

### 2.3 Psychiatric symptom assessment

A questionnaire was designed to collect demographic data. The severity of clinical symptoms was evaluated by the Positive and Negative Syndrome Scales (PANSS) by three experienced psychiatrists who had simultaneously attended a training session on the use of PANSS before the start of the study. After training, an inter‐rater intra‐class correlation coefficient with a total score greater than 0.8 was maintained by repeated assessments. At baseline and 4‐week follow‐up, PANSS was used to evaluate the severity of psychotic symptoms. The reduction rate in the PANSS score was calculated by the formula: (*T*_*b*_ − *T*_*a*_) /*T*_*a*_ × 100%, where *T*_*a*_ is the score at baseline and *T*_*b*_ is the score at follow‐up.^38^ Patients were defined as responders when the reduction rate of the PANSS total score was more than 30% according to previous studies.^39^, ^40^

### 2.4 Plasma collection and processing

At 7.00 a.m., fasting venous blood samples were collected from each patient both at baseline and 1‐month follow‐up. Plasma was separated and 200 μl of the sample was divided into two parts for extraction of water‐soluble and lipid‐soluble metabolites. For each part, 100 mg of the powder was transferred to a tube, to which 1 ml of methanol (for lipid‐soluble metabolites) or 75% (v/v) methanol (for water‐soluble metabolites) was added. The lipid‐soluble supernatant and water‐soluble supernatant were mixed (1:1), 0.6 ml of the mixture was dried, redissolved in 80 μl of 50% methanol, and filtered through a 0.1‐mm membrane in preparation for LC‐high resolution (HR)‐MS analysis.

### 2.5 Metabolomics data processing

A UPLC‐HRMS system, HRMS, and Q‐Exactive Focus equipped with a heated electrospray ionization source were used to profile the untargeted metabolomics. In this study, a scan in the mass range of 70–1000 m/z was acquired, at three scans per second with a resolution of 70,000. For the MS/MS assay, normalized collision energy of 35 V, isolation window of 0.8 m/z, and mass resolution of 35,000 were used. Principal component analysis (PCA) was performed using SIMCA‐P 13 software (Umetrics). A PLS‐DA (partial least squares discriminant analysis) model was used for the calculation of variable importance in projection (VIP) values. Annotated compounds were identified by searching the accurate mass of the molecular ions and fragment ions in compound databases. Metabolites were classified in HMDB or Lipidmap databases.

### 2.6 Statistical analysis

Demographic and clinical data between baseline and follow‐up were compared using SPSS Statistics 20.0. Although the variables in this study were normally distributed among the patients (Shapiro–Wilk test), the sample size was small; therefore, the primary outcome analysis consisted of a non‐parametric analysis for comparison between the two groups. Non‐parametric analysis of paired sample *t*‐tests was performed to compare the differences in the clinical symptoms before and after treatment with olanzapine for 4 weeks. Spearman rank correlation analysis was performed to analyze the association between the changes in the metabolites and the reduction rate of the PANSS scores. To obtain Bonferroni‐corrected/adjusted *p* values, we divided the original α‐value by the number of analyses performed on the variables. In this study, the new α = 0.05/72 = 0.00069 for metabolites and α = 0.05/4 = 0.0125 for psychotic symptoms. Further linear regression analysis was conducted to identify factors associated with improvement in psychotic symptoms, with the reduction rate of the PANSS total score as the dependent variable, and demographic data and changes in the metabolites as the independent variables. In the regression model, age and baseline body mass index (BMI) were added as the covariates, considering that education, onset age, and duration of illness were not associated with the KYN metabolites or the reduction rate of the clinical symptoms. In addition, logistic regression analysis was performed to evaluate the predictive utility of the identified KYN metabolites in discriminating between patients with good and poor response to olanzapine treatment.

## 3 RESULTS

### 3.1 Demographic and clinical data of patients

Table 1 shows the demographic and clinical data of the patients. Demographic and clinical characteristics were compared between the responders and non‐responders. We found that responders had higher PANSS G subscores than non‐responders (*p* < 0.05).

**TABLE 1:** Demographic and clinical variables of study participants at baseline

Variables	Responders	Non‐responders		Whole sample
	*n* = 10	*n* = 15	*z* (*p*)^a^	*n* = 25
Age (yrs)	27.9 ± 8.8	27.0 ± 7.0	0.31 (0.76)	27.4 ± 7.6
Education (yrs)	9.1 ± 3.8	9.1 ± 3.4	0.12 (0.91)	9.1 ± 3.5
BMI (kg/m^2^)	21.1 ± 3.9	20.9 ± 3.5	0.08 (0.93)	21.0 ± 3.6
Age of onset (yrs)	26.9 ± 9.9	26.0 ± 8.3	0.36 (0.72)	26.4 ± 8.8
PANSS total score	88.4 ± 8.9	77.4 ± 15.2	1.9 (0.06)	81.8 ± 13.9
P subscore	26.4 ± 4.7	24.1 ± 6.8	0.58 (0.56)	25.0 ± 6.0
N subscore	18.4 ± 6.3	16.2 ± 3.3	0.64 (0.52)	17.1 ± 4.7
G subscore	43.6 ± 5.1	37.1 ± 7.9	2.1 (0.04)	39.7 ± 7.5

Abbreviations: BMI, body mass index; G subscore, general psychopathology subscore; N subscore, negative symptom subscore; P subscore, positive symptom subscore; PANSS, Positive and Negative Syndrome Scale; yrs, years.

^a^ Independent samples *t*‐test (non‐parametric alternative Mann–Whitney *U*‐test).

After 4 weeks of olanzapine monotherapy, FEDN patients showed a statistically significant improvement in the psychotic symptoms, including positive subscore, general psychopathology subscore, and total score (all *p*_Bonferroni_ <0.05) (Table 2).

**TABLE 2:** Psychotic symptoms and MNFT levels at baseline and at follow‐up

	T1	T2	T2‐T1 (95% CI)	*z* (*p*)^a^
	*n* = 25	*n* = 25		
PANSS total score	84.4 ± 12.9	66.7 ± 13.5	−17.9 (−24.70, −11.06)	3.8 (<0.001)
P subscore	25.6 ± 5.2	16.9 ± 5.6	−8.6 (−10.98, −6.30)	4.2 (<0.001)
N subscore	18.2 ± 5.3	16.6 ± 4.0	−1.5 (−3.93, 0.97)	1.0 (0.30)
G subscore	40.6 ± 6.7	33.2 ± 5.7	−7.8 (−11.57, −3.95)	3.2 (0.001)
Metabolites				
MNFT	273,146.7 ± 210,146.7	335,688.1 ± 230,701.9	62,541 (−75,235, 200,318)	1.3 (0.21)
MNFT (responder)	166,878.8 ± 866,74.2	355,875.6 ± 282,535.8	188,996 (−19,252, 397,246)	1.9 **(**0.059)
MNFT (non‐responder)	343,991.9 ± 239,650.6	322,229.8 ± 198,589.7	−21,762 (−211,563, 168,038)	0.06 (0.96)
Lg (MNFT levels)				
MNFT	5.3 ± 0.3	5.4 ± 0.3	0.09 (−0.10, 0.28)	1.2 (0.24)
MNFT (responder)	5.2 ± 0.2	5.4 ± 0.3	0.26 (−0.20, 0.54)	1.8 (0.07)
MNFT (non‐responder)	5.4 ± 0.3	5.4 ± 0.3	−0.02 (−0.28, 0.25)	0.0 (1.00)

Abbreviations: MNFT, methyl n‐formylanthranilate; T1, at baseline; T2, at 4‐week follow‐up; T2−T1, the value in T2 minus the value in T1.

^a^ Paired samples *t*‐test (non‐parametric alternative Mann–Whitney *U*‐Test).

### 3.2 Metabolomic analysis at the baseline and after 4‐week treatment

As described in our previous study, we found 4299 differential compounds with *p* < 0.05 and 1280 compounds with VIP > 1. After screening with *p* < 0.05 and VIP > 1, 175 compounds were identified in the differential compound library by mass spectrometry. Finally, 72 differential compounds were identified and classified in HMDB or Lipidmap databases, as reported in our previous study.^29^ In our previous study, we also reported a significant association between plasma lysophosphatidylcholine or lysophosphatidylethanolamine and therapeutic response to olanzapine monotherapy in FEDN patients with SZ.^41^ In the present study, a differential compound in the tryptophan metabolic pathway, methyl n‐formylanthranilate (MNFT) (chemical formula: C9H9NO3; HMDB0032398; Molecular weight: 179.1727), was analyzed, and we found a trend toward statistically significant differences in the MNFT levels in responders between the baseline and 4‐week follow‐up (*z* = −1.9, *p* = 0.059). MNFT levels were slightly increased in responders after 4 weeks of treatment compared to the baseline levels.

### 3.3 Association between the baseline metabolites and reduction rate in the PANSS score

**FIGURE 1:**
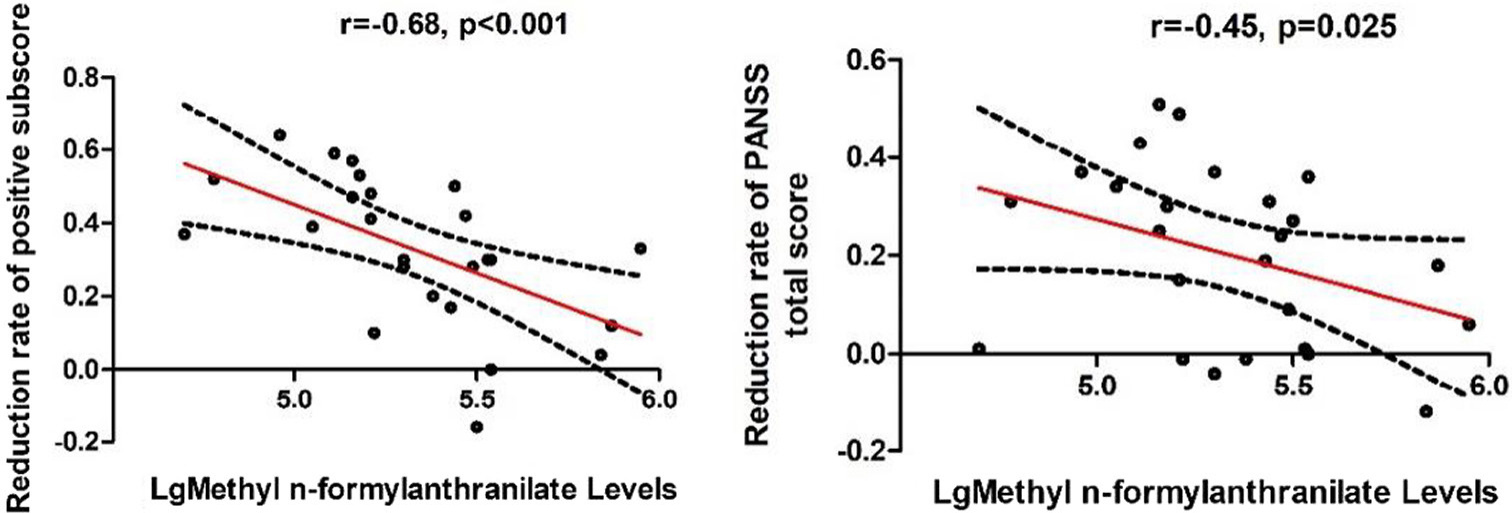
There were negative associations between the baseline Methyl n‐formylanthranilate levels and the reduction rate in the positive subscore or PANSS total score in FEDN patients after 4 weeks of olanzapine monotherapy (all *p* < 0.05). The red line is the trend line and the black dashed line is the 95% confidence interval

Spearman rank correlation analysis revealed that the baseline MNFT levels were correlated with the reduction rate in the positive subscore (*r* = −0.68, *p* < 0.001) or PANSS total score (*r* = −0.45, *p* = 0.025) (Figure 1). However, only the significant association between MNFT levels and reduction in the positive subscore passed the Bonferroni correction (*p* > 0.05). Multiple regression analysis with the baseline MNFT level as the dependent variable, and age and baseline BMI as the covariates showed a significant association between the baseline MNFT level and reduction rate of the positive subscore after treatment with olanzapine for 1 month (β = −0.63, *t* = −3.74, *p* = 0.001), which accounted for 37.0% of the variance. In addition, there was a significant correlation between the reduction rate of PANSS total score and baseline MNFT levels (β = −0.42, *t* = −2.08, *p* = 0.05).

### 3.4 Predictive ability of baseline MNFT for the treatment response

There was a significant difference in the baseline MNFT levels between responders and non‐responders (*z* = 2.39, *p* = 0.017). Further logistic regression analysis was performed with response to treatment as the dependent variable, baseline MNFT level as the independent variable, and BMI at baseline and age as the covariates. We found that the baseline MNFT levels were predictive of the response to olanzapine after 4‐week treatment (β = −3.9, Wald *X* ^2^ = 4.0, *p* = 0.046, odds ratio [OR] = 0.021).

Moreover, receiver operating characteristic curve (ROC) analysis revealed that the area under the ROC curve (AUC) value of the baseline MNFT levels for the therapeutic response was 0.79. According to previous studies, a concordance statistic between 0.7 and 0.8 is generally considered acceptable.^42^, ^43^

### 3.5 Association between the changes in plasma MNFT and improvement in symptoms

A correlation analysis was performed to determine whether the changes in the MNFT levels were correlated with the reduction rate of PANSS score after olanzapine monotherapy. We found that an increase in the MNFT levels was not associated with the reduction rate of the PANSS total score or its subscores (all *p* > 0.05).

## 4 DISCUSSION

This study has several important findings. (1) We found that the baseline MNFT levels were associated with the reduction rate of positive subscore after olanzapine monotherapy in FEDN patients. (2) Baseline MNFT level may serve as a predictor of the therapeutic response to olanzapine. (3) Reduced PANSS scores after treatment were not associated with altered MNFT levels.

To our best knowledge, this is the first study to identify a close relationship between the baseline MNFT level and reduction in the PANSS score after treatment with olanzapine. MNFT belongs to a class of organic compounds known as acylaminobenzoic acid derivatives (https://hmdb.ca/metabolites/HMDB0032398). MNFT is involved in the indole degradation pathway used by gram‐negative bacteria.^44^ It also plays a key role in the KYN pathway of tryptophan metabolism and participates in the metabolism of formylanthranilate to N‐formylanthranilate.^45^ Several key components in the tryptophan metabolism pathway have been found to be dysregulated in SZ,^20^ and have been implicated in the pathogenesis and pathophysiology of SZ.^19^, ^46^, ^47^ Considering the close relationship between MNFT and the KYN pathway, we speculate that the association between MNFT levels and symptom improvement may be an indirect consequence of the abnormal KYN pathway in SZ.

Our finding of an association between the baseline MNFT levels and improvement in clinical symptoms and response to olanzapine monotherapy in FEDN patients with SZ provides further evidence for the role of an abnormal KYN pathway in the pathogenesis and treatment of SZ. Moreover, we found that the baseline MNFT levels were lower in responders compared with non‐responders, but there was no significant difference in the MNFT levels between responders and non‐responders after treatment with olanzapine. Olanzapine treatment slightly increased the MNFT levels in responders compared with non‐responders; however, we could not make a comparison with other studies, since this is the first study to investigate the role of MNFT in the treatment response to olanzapine in SZ. However, our results were consistent with previous findings on the KP pathway in SZ.^48^ For example, recent evidence from clinical studies suggests that KP pathway molecules in plasma are associated with those in the central nervous system, and may be potential novel biomarkers to monitor progression after treatment with antipsychotics in patients with SZ.^20^, ^49^, ^50^, ^51^

We found that a lower baseline level of MNFT was associated with better response to treatment and MNFT was elevated in responders at the 4‐week follow‐up. This suggests that MNFT levels are dynamic from baseline to post‐treatment and olanzapine may regulate the levels. However, we do not know the exact reason for the changes that occur at the baseline and at the end of the treatment, since the exact function of MNFT in the body remains unclear. However, it is closely related to the kynurenines (KYNU) in the KP pathway, and the post‐treatment changes indirectly indicate that the dysregulated KP pathway in patients with SZ can be elaborately regulated by olanzapine. We speculate that olanzapine treatment increased the KYNU enzyme activity in SZ patients, which in turn led to increased MNFT levels in responders. MNFT level may be a more sensitive indicator of olanzapine treatment in the KP pathway in the early stage of SZ. There is mounting evidence regarding the inflammatory processes and glutamatergic dysfunction involved in the pathophysiology of SZ.^52^, ^53^, ^54^, ^55^, ^56^, ^57^, ^58^, ^59^ Mechanically, the KP pathway is involved in immunomodulation and brain development and has neurotoxic and neuroprotective effectors.^60^ Given its immunosuppressive properties, the KP pathway has been proposed as a counter‐regulatory mechanism in the glutamatergic system and inflammatory response.^61^ Another possible explanation is that olanzapine treatment may alter the binding affinity of the KYNU enzyme and MNFT, possibly leading to altered renal clearance of MNFT and increased MNFT levels. Altogether, our findings provide new evidence on the potential prognosis biomarkers and treatment targets in the KP pathway in SZ. However, we did not find a statistically significant association between increased MNFT levels and the reduction rate of the PANSS total score. Further investigation of the relationship between MNFT levels and the therapeutic response to olanzapine in SZ in a large sample is warranted.

The present study has several limitations. First, the relatively small sample size increased the likelihood of a Type II error, which can decrease the statistical power. Several of the analyses presented did not produce statistically significant findings, which might be due to Type II errors. For example, the finding of a trend toward a statistically significant difference in the MNFT levels between baseline and follow‐up may be due to the small sample size. Second, only female patients were recruited in this study, and the findings may not be generalized to male patients. Third, future studies should recruit control subjects and evaluate other antipsychotics to verify the clinical value of MNFT in predicting the treatment response to different types of antipsychotics. Fourth, the exact function of MNFT is still unclear. This study can provide little insight into the exact molecular mechanism underlying the close relationship between MNFT and response to olanzapine treatment in SZ. Fifth, in this study, we did not use structured interviews or psychometric tools to confirm that the participants were not alcohol dependent. We only confirmed verbally whether the patients drank alcohol or not, considering that all patients were hospitalized during the study and were not permitted to drink alcohol. Sixth, since the study was conducted solely on the Han Chinese population, this might limit the generalization of the findings to the global population.

In summary, we found that the baseline levels of MNFT were associated with the reduction rate in the PANSS positive subscore and response to olanzapine monotherapy for 4 weeks. Considering that MNFT is a key metabolite in the KP pathway and is closely related to the KYNU enzyme, our findings provide further evidence for the role of the KYN pathway in the pathogenesis and treatment of SZ. Nevertheless, given that the sample was limited to women and was relatively small, great caution is needed in interpreting our findings. Further longitudinal studies with larger numbers of patients treated with different types of antipsychotics are warranted.

## AUTHOR CONTRIBUTIONS

XG, XS, JX, and HL were responsible for clinical data collection. JX and XW were responsible for the laboratory experiments. HL and FW were involved in evolving the ideas and editing the manuscript. HL, RL, and FW were responsible for study design, statistical analysis, and manuscript preparation. All authors have contributed to and approved the final manuscript.

## CONFLICT OF INTEREST

The author(s) declared no potential conflicts of interest with respect to the research, authorship, and/or publication of this article.

## Data Availability

The data that support the findings of this study are available from the corresponding author upon reasonable request.
